# Glioma Grade Classification Using Machine Learning and MRI Radiomics: A Single‐Center Prospective Study Comparing Original and Wavelet‐Transformed Features From Anatomical, Diffusion‐Weighted, and Post‐Contrast Imaging

**DOI:** 10.1002/hsr2.72995

**Published:** 2026-08-11

**Authors:** Amir Khorasani, Daryoush Shahbazi‐Gahrouei

**Affiliations:** ^1^ Department of Bioimaging, School of Advanced Technologies in Medicine Isfahan University of Medical Sciences Isfahan Iran; ^2^ Medical Image and Signal Processing Research Center, School of Advanced Technologies in Medicine Isfahan University of Medical Sciences Isfahan Iran; ^3^ Department of Medical Physics, School of Medicine Isfahan University of Medical Sciences Isfahan Iran

**Keywords:** classification, diffusion‐weighted imaging, Glioma, machine learning, random forest

## Abstract

**Background and Aims:**

Accurate glioma grade classification is critical for prognostic assessment and clinical decision‐making. This study aimed to evaluate the impact of wavelet‐based radiomics feature analysis on the performance of machine learning (ML) models for glioma grade classification using diffusion‐weighted imaging (DWI), structural MRI sequences, and contrast‐enhanced T_1_‐weighted (T_1_Gd) images.

**Methods:**

This prospective study included 92 patients with histopathologically confirmed gliomas. MRI examinations were performed using a 1.5 T scanner and included apparent diffusion coefficient (ADC) and exponential ADC (eADC) maps, T_1_‐weighted, T_2_‐FLAIR, and T_1_Gd sequences. Image preprocessing consisted of deep learning–based autoencoder denoising and intensity normalization using the Removal of Artificial Voxel Effect by Linear regression (RAVEL) method. Tumor volumes of interest were manually segmented by two experienced neuroradiologists. Radiomics features were extracted from original images and wavelet‐transformed images by Pyradiomics, followed by feature selection using the least absolute shrinkage and selection operator (LASSO). Glioma grade classification was performed using fine‐tuned Decision Tree, Logistic Regression, K‐nearest neighbors, Naïve Bayes, Support Vector Machine, Multi‐layer Perceptron, and Random Forest models, with hyperparameters tuned via fivefold cross‐validation on an 80% training subset and performance assessed on an independent 20% held‐out test set.

**Results:**

The addition of wavelet‐based radiomics features significantly improved classification performance across all ML models and MRI sequences. The highest performance was achieved by a fine‐tuned Random Forest model trained with original and wavelet‐transformed images' radiomics features extracted from eADC maps, with an average AUC of 0.96, accuracy of 0.97, sensitivity of 0.94, and specificity of 0.91.

**Conclusion:**

Adding wavelet‐transformed radiomics feature to original image radiomics feature analysis of eADC maps combined with fine‐tuned Random Forest classification provides an accurate, non‐invasive, and efficient approach for glioma grade classification with strong potential for clinical application.

## 1 Introduction

Gliomas are the most common primary brain tumors, originating from glial cells [1]. They are increasingly recognized as a significant global public health concern due to their high morbidity and mortality. Accurate detection and effective management of gliomas remain major challenges in medical and neuroscientific research [1]. Treatment strategies for glioma are determined based on clinical presentation and intrinsic tumor characteristics, among which tumor grade is one of the most critical factors [1]. Glioma grading plays a central role in guiding therapeutic decision‐making and prognostic evaluation [2]. According to the World Health Organization (WHO) classification system, gliomas are stratified into low‐grade gliomas (LGGs) and high‐grade gliomas (HGGs) based on histopathological features, biological behavior, and tumor aggressiveness [3].

The current gold standard for glioma grade classification is based on histopathological findings. However, the significant problems with this kind of application are tissue sampling error during the biopsy, the invasive nature of the procedure, and significant intra‐observer variation [4, 5, 6]. Recently, researchers have shown an increased interest in using non‐invasive methods for glioma‐grade detection [4, 5, 6]. Advancements in medical imaging, particularly in magnetic resonance imaging (MRI), have sparked renewed interest in utilizing MRI scans for glioma grading. Much literature has been published on using different MRI image weights for glioma grade classification [4, 5, 6, 7]. One of the main issues in our knowledge of using MRI image weights for glioma grading is a lack of a highly accurate grade classification of MRI image weights. One of the most significant current discussions in glioma grading is using Artificial Intelligence (AI) [7, 8].

Machine Learning (ML) is a significant area of interest in using AI in glioma grade classification [8]. Recent trends in using ML for glioma grading have led to a proliferation of studies that used radiomics, a specific method for medical image analysis and image feature extraction [9]. The emergence of radiomics allows for the extraction of quantitative image parameters, which can be applied to medical purposes, including glioma grading. Mathematical algorithms may be used to extract various quantitative information from medical images using radiomics as an advanced image processing model. The fundamental tenet of the radiomics endeavor is that imaging characteristics may identify important phenotypic variations in malignancies and be used for diagnosis, prognosis, and outcome prediction. Recent evidence suggests that Wavelet Transform (WT) sub‐band image features can be used for radiomics analysis [10].

A large and growing body of literature has investigated radiomics analysis for glioma grade classification with different ML algorithms [11, 12, 13, 14]. Most studies in this field have only focused on using structural MRI image weights such as T_1_, T_2_, and T_1_ post‐contrast (T_1_Gd) [13, 14, 15]. Previous research [12] has indicated that various MRI image weights, such as Diffusion‐weighted imaging (DWI), perfusion MRI, etc., positively impact glioma grade classification performance with ML. This paper will use an Apparent diffusion coefficient map (ADC map) and an Exponential ADC map (eADC map) of DWI for glioma grade classification by radiomics analysis. No previous study has investigated the impact of the WT sub‐band image feature of ADC map and eADC map for glioma grade classification by radiomics analysis. The principal objective of this study was to investigate the impact of the WT sub‐band image feature of ADC map, eADC map, T_1_, T_2_, and T_1_Gd for glioma grading with different ML algorithm and introduce the highest performance configuration for posible clinical usage.

## 2 Material and Methods

This study was conducted as a prospective study and was designed in several sequential stages, including patient selection, MRI image acquisition, image preprocessing, feature extraction, classification using ML models, and model evaluation.

### 2.1 Pateint Selection

Histopathological diagnosis following surgical tumor resection was used as the ground truth for glioma grading in this study. All patients underwent MRI examinations prior to surgery and before the initiation of radiotherapy or chemotherapy. Tumors were classified into low‐grade glioma (LGG) and high‐grade glioma (HGG) based on histopathological evaluation. Detailed stratification according to individual WHO grades (II, III, and IV), tumor subtypes, and molecular markers was not performed, as the primary objective of this study was binary glioma grade classification. Patients were enrolled according to inclusion and exclusion criteria. Eligible participants had histopathological confirmation of glioma grade, had undergone MRI examinations before any therapeutic intervention (including surgery, chemotherapy, or radiotherapy), and had high‐quality MRI images without significant artifacts. Patients were excluded if they were pregnant, had a known allergy to gadolinium‐based contrast agents, or experienced claustrophobia that prevented completion of MRI scanning. The study protocol was approved by the Local Research Ethics Board and the Research Ethics Committee of Isfahan University of Medical Sciences, Isfahan, Iran (Approval ID: IR. ARI. MUI. REC.1403.054). Written informed consent was obtained from all participants prior to enrollment, and after consent was secured, all participants underwent MRI scanning, and the required imaging sequences were acquired for the purposes of this study.

### 2.2 MRI Imaging

All patients were examined using a clinical 1.5 Tesla MRI scanner (GE SIGNA Explorer 1.5 T). The imaging protocol included diffusion‐weighted imaging (DWI) sequences with corresponding ADC and eADC maps, as well as T_1_‐weighted, T_2_‐FLAIR, and contrast‐enhanced T_1_‐weighted (T_1_Gd) sequences. Detailed acquisition parameters for each sequence are provided in Table 1. The DWI, T_1_‐weighted, and T_2_‐FLAIR images were obtained prior to the administration of the gadolinium‐based contrast agent. For consistency in subsequent analysis, all acquired images were resized to a spatial resolution of 256 × 256 pixels.

**Table 1:** Magnetic resonance imaging (MRI) pulse sequences parameters.

Image weights	TR (ms)	TE (ms)	FOV (mm)	b‐value (s/mm2)	Matrix size	Thickness (mm)	Gap (mm)
ADC map	5211	110	240 × 240	50–1000	256 × 256	5	5
eADC map	5211	110	240 × 240	50–1000	256 × 256	5	5
T_2_‐FLAIR	8500	97.05	240 × 240	—	512 × 512	5	5
T_1_	400	10	240 × 240	—	512 × 512	5	5
T_1_Gd	6.1	2.2	240 × 240	—	512 × 512	5	5

Abbreviations: ADC, apparent diffusion coefficient; eADC, exponential ADC; FOV, field of view; TE, time of echo; TR, time of repetition.

### 2.3 Image Preprocessing

**Figure 1:**
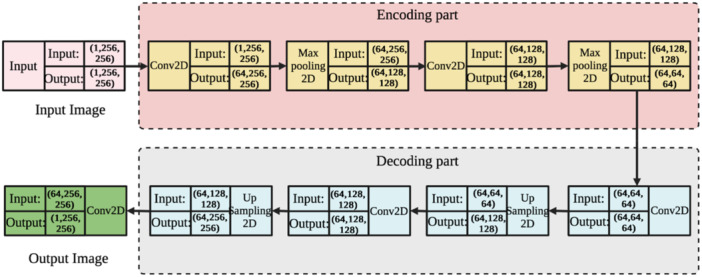
This study employs an autoencoder architecture for image denoising. Each box represents distinct operations, including convolution, max‐pooling, and upsampling layers, with the input and output feature map sizes indicated on each box.

Medical image pixel intensities are influenced by tissue properties, noise, and scanner‐related artifacts. To enhance image quality and improve the robustness of subsequent image analysis, normalization and denoising were applied during the preprocessing stage. Previous studies have demonstrated that both normalization and denoising can substantially affect analysis outcomes and are therefore essential preprocessing steps in medical image analysis [16, 17, 18, 19]. In this study, a deep learning–based denoising approach was implemented using a convolutional autoencoder architecture (Figure 1), while intensity normalization was performed using the Removal of Artificial Voxel Effect by Linear regression (RAVEL) method. The denoising and normalization procedures followed the methodologies described in prior work [16, 20], and further methodological details can be found in these references. Because radiomics features were extracted specifically from predefined volumes of interest (VOIs), skull stripping was not expected to influence the extracted features; therefore, skull stripping [21] was not performed in this study. The autoencoder was trained from scratch for this study using a dataset of normal‐appearing brain tissue 2D slices. This approach ensures that the denoising transformation is independent of the subsequent tumor classification task and introduces no label‐related information leakage. All preprocessing procedures were implemented using custom‐developed code written in Python.

### 2.4 Tumor Segmentation and Mask Creation

After image preprocessing, all images were imported into 3D Slicer software (version 4.11) for mask creation and radiomics analysis. Tumor regions of interest (ROIs) were manually delineated on each MRI sequence by two independent board‐certified neuroradiologists, each with more than 16 years of experience in glioma diagnosis. Gliomas are known to consist of heterogeneous subregions, including peritumoral edema, necrotic core, and active (enhancing) tumor tissue. In this study, segmentation was performed to primarily include the active tumor region and necrotic, and, where feasible, areas of edema, and hemorrhage were excluded to ensure consistency of feature extraction. For each patient, the final VOIs were generated from the slice‐by‐slice ROIs and were subsequently used for radiomics feature extraction. The spatial consistency between the two sets of segmentations was quantified using the Dice Similarity Coefficient (DSC).

### 2.5 Feature Extraction

Radiomics feature extraction was performed using the PyRadiomics library (https://pyradiomics.readthedocs.io/en/latest/) to obtain a comprehensive set of quantitative features from the segmented VOIs. The PyRadiomics framework enables standardized and reproducible feature extraction through configurable feature classes, image types, and extraction parameters. In this study, features were extracted from two image types: the original images and wavelet‐transformed images, where wavelet decomposition applies multi‐scale filtering to emphasize textural information at different spatial frequencies. The extracted feature classes included shape features, first‐order statistics, Gray Level Co‐occurrence Matrix (GLCM), Gray Level Dependence Matrix (GLDM), Gray Level Run Length Matrix (GLRLM), Gray Level Size Zone Matrix (GLSZM), and Neighborhood Gray‐Tone Difference Matrix (NGTDM) features. Key extraction parameters were set to a bin width of 25, interpolator = sitkBSpline, and defined resampled pixel spacing. A complete list of all extracted radiomics features is provided in Supplementary 1. For detailed methodological and computational descriptions of PyRadiomics feature definitions, readers are referred to the PyRadiomics documentation and the original reference [22].

### 2.6 Feature Selection

To minimize the influence of differences in feature magnitudes, Min–Max normalization was applied to all extracted features, rescaling their values to a common range from 0 to 1, thereby improving the stability and comparability of subsequent analyzes. In the previous stage, many features will be extracted from VOIs. Many of them are just noise or are highly correlated with each other. So, it was necessary to select the most essential features. Improper selection of features can increase the probability of overfitting the networks, increase computational cost and time, and decrease prediction accuracy. For feature selection, we used the Least Absolute Shrinkage and Selection Operator (LASSO) method in this study to select the most critical features for glioma‐grade classification. Feature selection was performed exclusively on the training subset of the patient‐wise train–test split described in Section 2.7 that the independent test set remained fully unseen at this stage. LASSO‐based feature selection was applied once to the entire training set, prior to and separately from the subsequent hyperparameter‐tuning cross‐validation loop described in Section 2.7.

### 2.7 ML and Classifier Models

This study classified glioma into HGGs and LGGs with different ML methods. Glioma grade classification was performed using Decision Tree (DT), Logistic Regression (LR), K‐nearest neighbors (KNN), Naïve Bayes (NB), Support vector machines (SVM), Multi‐layer Perceptron (MLP), and Random Forest (RF) ML algorithms. We used Python's ‘scikit‐learn’ package (Version 1.8.0) to create MLs algorithms.

The dataset was randomly divided into training (80%) and independent testing (20%) subsets using the “train_test_split” function. The test set was held out and not used during model training, feature selection, or hyperparameter optimization. Within the training set, a fivefold cross‐validation strategy was employed to fine‐tune model hyperparameters. The primary performance metrics reported in this study (AUC, accuracy, sensitivity, and specificity) were obtained by applying the optimized, fine‐tuned models to this independent, held‐out test set. A separate repeated cross‐validation analysis, described in Section 2.8, was additionally performed to statistically compare the effect of wavelet‐transformed radiomics features and was not used to generate the primary reported performance metrics.

Hyperparameter optimization was performed using grid search with stratified fivefold cross‐validation (GridSearchCV, scikit‐learn). Separate grids were defined for each classifier, including DT, LR, KNN, NB, SVM, MLP, RF with hyperparametres in Table 2. The optimal hyperparameters were selected based on the highest mean cross‐validation performance.

**Table 2:** Hyperparameter search space used for grid search optimization of all machine learning classifiers. Optimal values were selected using stratified k‐fold cross‐validation.

Model	Hyperparameter	Range/Values tested
Decision Tree (DT)	max_depth	None, 5, 10, 20, 30
	min_samples_split	2, 5, 10
	min_samples_leaf	1, 2, 5
	criterion	gini, entropy
Logistic Regression (LR)	C	0.01, 0.1, 1, 10, 100
	solver	liblinear, lbfgs
K‐Nearest Neighbors (KNN)	n_neighbors	3, 5, 7, 9, 11
	weights	uniform, distance
	metric	euclidean, manhattan
Naïve Bayes (NB)	var_smoothing	1e − 12, 1e − 9, 1e − 6, 1e − 3
Support Vector Machine (SVM)	C	0.1, 1, 10, 100
	kernel	linear, rbf
	gamma	scale, 0.01, 0.001
Multi‐layer Perceptron (MLP)	hidden_layer_sizes	(50), (100), (50,50), (100,50)
	activation	relu, tanh
	alpha	0.0001, 0.001, 0.01
	learning_rate	constant, adaptive
Random Forest (RF)	n_estimators	100, 200, 500
	max_depth	None, 10, 20, 30
	min_samples_split	2, 5, 10
	min_samples_leaf	1, 2, 4

### 2.8 Model's Training, Result Evaluation, and Statistical Test

As a complementary analysis to the train‐test evaluation described in Section 2.7, and specifically to enable a paired statistical comparison between feature configurations (i.e., original vs. original plus wavelet‐transformed radiomics features), a repeated cross‐validation strategy was additionally employed. In this analysis, the entire dataset was evaluated using fivefold cross‐validation repeated 10 times with different random fold partitions. This repeated cross‐validation procedure was used solely to generate the paired performance estimates required for the Wilcoxon signed‐rank test described below and did not contribute to the primary performance metrics reported in the study. In each repetition, the data were split into five folds, with four folds used for model training and one fold used for validation. Feature selection and hyperparameter optimization were performed exclusively within the training folds to prevent data leakage.

The Accuracy (ACC), Sensitivity, Specificity, and receiver operating characteristic (ROC) curve analysis of the “metrics” subpackage of “scikit‐learn” were used to evaluate different MLs algorithm results on the test dataset. The impact of wavelet‐transformed radiomics features on model performance was assessed using a paired, non‐parametric statistical approach. Performance metrics were obtained from identical cross‐validation folds within the training dataset. For each ML algorithm and MRI sequence, metrics derived from models trained using original radiomics features were paired with those from models trained using combined original and wavelet‐transformed radiomics features. Paired comparisons were performed using the Wilcoxon signed‐rank test, which is appropriate for fold‐wise paired data and does not require assumptions of normality. A two‐sided *p* value < 0.05 was considered statistically significant. The association between patient sex and tumor grade was analyzed using Fisher's exact test, while the relationship between age and tumor grade was evaluated using the appropriate statistical test based on data distribution. In this study, *p* < 0.05 were indicated to be statistically significant. Statistical significance was analyzed using SPSS 26.0 software (IBM Corp., Armonk, NY, USA).

## 3 Results

**Figure 2:**
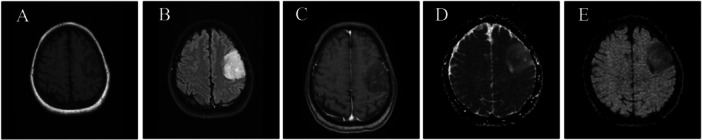
The images of a 44‐year‐old woman with low‐grade glioma (LGG). (A) T1, (B) T2‐FLAIR, (C) T1Gd, (D)‐ADC map, (E) eADC map.

Of 101 initially identified cases, 9 cases were excluded from the analysis. Specifically, one case was excluded due to imaging artifacts, one case was excluded because of inadequate image quality, and seven cases were excluded due to issues in tumor mask creation. Consequently, a total of 92 subjects with confirmed glioma were included in the radiomics analysis for glioma grade classification. Almost two‐thirds of the participants (64%) are HGGs, and one‐third (36%) are LGGs. There were no significant differences between age, sex, and glioma grade. The MRI images of a 44‐year‐old woman with LGG are shown in Figure 2.

**Figure 3:**
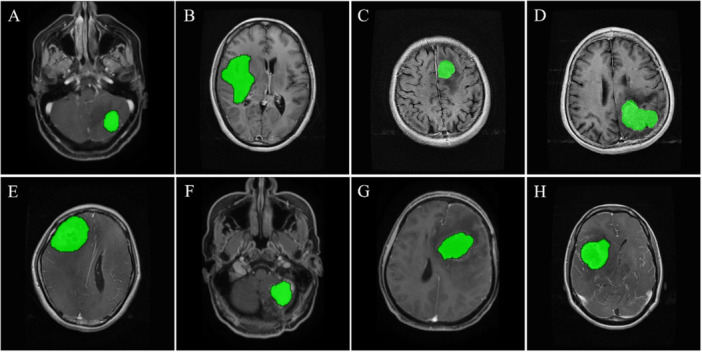
Representative contrast‐enhanced T1‐weighted (T1Gd) MRI images from randomly selected patients with high‐grade gliomas (HGGs). The manually tumor regions of interest (ROIs), used to generate volumes of interest (VOIs) for radiomics feature extraction, are overlaid in green.

Figure 3 illustrates representative T_1_Gd images from randomly selected patients diagnosed with HGG cases. The manually segmented tumor regions, highlighted in green, demonstrate the ROIs used to construct VOIs for radiomics feature extraction. The DSC was calculated between the VOIs segmented by Neuroradiologist A and Neuroradiologist B for all subjects. The mean (± standard deviation) DSC was 0.87 ± 0.07, indicating very good spatial consistency.

Radiomics feature extraction was performed on five MRI sequences, including T_1_‐weighted, T_2_‐FLAIR, T_1_Gd, ADC map, and eADC maps, using both conventional and wavelet‐based feature domains. For each MRI sequence, 107 conventional radiomics features and 851 conventional plus wavelet‐based features were initially extracted. Following feature selection using the LASSO, a substantially reduced subset of features was retained for subsequent model development. Among the conventional feature sets, the number of selected features ranged from 15 for T_1_‐weighted images to 29 for eADC maps, whereas for wavelet‐based features, the number of selected features ranged from 22 for T_2_‐FLAIR images to 41 for ADC maps. Overall, wavelet‐based feature domains yielded a larger number of selected features across all MRI sequences compared with conventional feature domains, highlighting their enhanced contribution to glioma grade discrimination. These results are summarized in Table 3.

**Table 3:** Summary of the number of radiomics features extracted and selected using the LASSO across different MRI sequences, including conventional and wavelet‐based feature domains.

MRI image weights	Feature domain	Nember of extracted features	Number of selected features with LASSO
T_1_	CON	107	15
	CON + WT	851	23
T_2_‐FLAIR	CON	107	18
	CON + WT	851	22
T_1_Gd	CON	107	25
	CON + WT	851	31
ADC map	CON	107	24
	CON + WT	851	41
eADC map	CON	107	29
	CON + WT	851	37

Abbreviations: CON, conventional; WT, Wavelet‐based.

**Figure 4:**
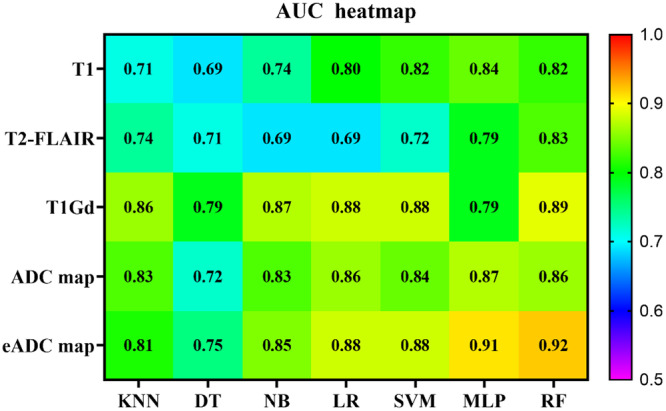
Average AUC Heat map of glioma grade classification with different fine‐tuned Machine Learning algorithms and MRI image weights with Conventional radiomcs features (without Wavelet‐based features). DT, Decision Tree; KNN, K‐nearest neighbors; LR, Logistic Regression; MLP, Multi‐layer Perceptron; NB, Naïve Bayes; RF, Random Forest; SVM, Support vector machines.

After feature selection, multiple ML classifiers were trained for HGG/LGG classification using fine‐tuned model parameters. All reported performance metrics in the results section, including those presented in tables and figures, correspond to the evaluation of fine‐tuned ML models on the independent test set. Figure 4 presents the average AUC heat map illustrating the performance of different fine‐tuned ML algorithms across various MRI sequences using conventional radiomics features (without wavelet‐based features). Notably, the fine‐tuned RF classifier (with parameters of n_estimators = 100, max_depth = 20, min_samples_split = 2, min_samples_leaf = 2) trained on features extracted from the eADC map achieved the highest AUC values among all evaluated combinations, indicating superior performance for glioma grade classification using conventional radiomics features extracted from this imaging modality.

**Figure 5:**
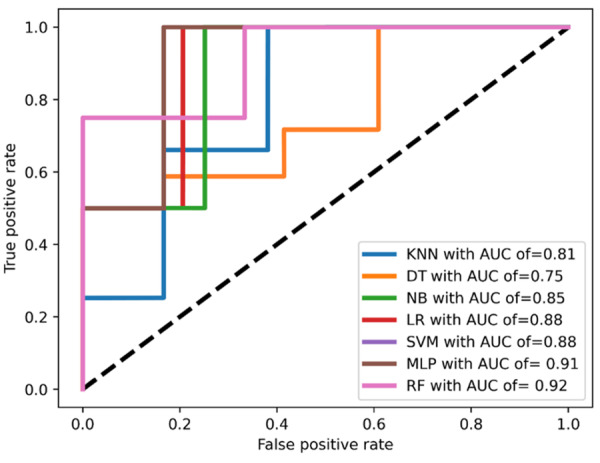
ROC curves of different fine‐tuned Machine Learning algorithms for glioma grade classification by eADC map images with Conventional radiomcs features (without Wavelet‐based features). DT, Decision Tree; KNN, K‐nearest neighbors; LR, Logistic Regression; MLP, Multi‐layer Perceptron; NB, Naïve Bayes; RF, Random Forest; SVM, Support vector machines.

The ROC curves for the fine‐tuned ML algorithms applied to glioma grade classification, using conventional radiomics features (without wavelet‐based features) of eADC maps, are presented in Figure 5. As shown, the fine‐tuned MLP (with parameters of hidden_layer_sizes = (50,50), activation = relu, alpha = 0.001, learning_rate = adaptive) and fine‐tuned RF (with parameters of n_estimators = 100, max_depth = 20, min_samples_split = 2, min_samples_leaf = 2) classifiers achieved the highest AUC values, with AUC values of 0.91 and 0.92, respectively, outperforming the other models evaluated in this study.

**Figure 6:**
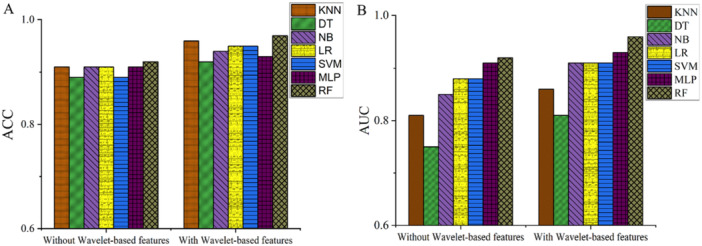
Impact of wavelet transformation radiomics features on the average (A) accuracy and (B) area under the ROC curve (AUC) for glioma grade classification using radiomics features extracted from eADC maps across different machine learning algorithms. DT, Decision Tree; KNN, K‐nearest neighbors; LR, Logistic Regression; MLP, Multi‐layer Perceptron; NB, Naïve Bayes; RF, Random Forest; SVM, Support Vector Machine.

**Figure 7:**
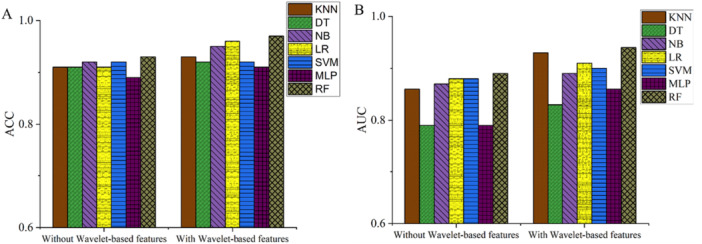
Impact of wavelet transformation radiomics features on the average (A) accuracy and (B) area under the ROC curve (AUC) for glioma grade classification using radiomics features extracted from T1Gd across different machine learning algorithms. DT, Decision Tree; KNN, K‐nearest neighbors; LR, Logistic Regression; MLP, Multi‐layer Perceptron; NB, Naïve Bayes; RF, Random Forest; SVM, Support Vector Machine.

Figures 6 and 7 illustrate the effect of incorporating wavelet‐based radiomics features on the average classification ACC and AUC of the fine‐tuned ML algorithms for glioma grade classification using eADC maps and T_1_Gd images, respectively. Notably, the inclusion of wavelet‐based features resulted in substantial improvements in both ACC and AUC across the evaluated models. Overall, the use of wavelet‐based radiomics features for ML training and testing led to statistical significant increases in ACC, AUC, sensitivity, and specificity across all MRI sequences and ML algorithms considered in this study.

**Figure 8:**
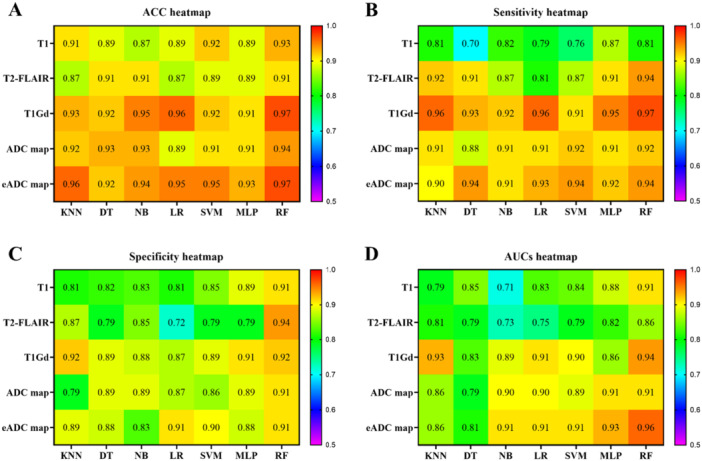
Glioma grade classification heat maps with different Machine Learning algorithms and MRI image weights with Wavelet transform. (A) average Accuracy heat map, (B) average Sensitivity heat map, (C) average Sensitivity heat map, (D) average AUC heat map. DT, Decision Tree; KNN, K‐nearest neighbors; LR, Logistic Regression; MLP, Multi‐layer Perceptron; NB, Naïve Bayes; RF, Random Forest; SVM, Support vector machines.

The average of performance metrics, ACC, AUC, sensitivity, and specificity, for HGG/LGG classification using conventional radiomics features adding with wavelet‐based features are summarized in the heat maps presented in Figure 8. Crucially, the fine‐tuned RF classifier (with parameters of n_estimators = 200, max_depth = 20, min_samples_split = 2, min_samples_leaf = 2), trained on wavelet features extracted from eADC maps, demonstrated superior performance, achieving the highest aggregate metrics among all models evaluated with an AUC of 0.96 (95% CI: 0.92–0.99), accuracy of 0.97 (95% CI: 0.93–0.99), sensitivity of 0.94 (95% CI: 0.88–0.98), and specificity of 0.91 (95% CI: 0.83–0.97). This represents the most significant finding of our study.

To complement the heat map visualizations above, Table 4 summarizes the performance of the fine‐tuned Random Forest classifier, the best‐performing model in this study, across all five MRI sequences, with and without wavelet‐transformed radiomics features, along with the corresponding 95% confidence intervals. Consistent with the heat maps, the addition of wavelet‐based features improved all four performance metrics for every MRI sequence evaluated, with the largest relative gain observed for T_1_‐weighted images, where sensitivity increased from 0.61 (95% CI: 0.57–0.71) to 0.81 (95% CI: 0.77–0.84).

**Table 4:** Performance of the fine‐tuned Random Forest classifier for glioma HGG/LGG classification across different MRI sequences, using conventional (CON) and conventional plus wavelet‐transformed (CON + WT) radiomics features, with 95% confidence intervals.

MRI sequence	Feature set	AUC (95% CI)	Accuracy (95% CI)	Sensitivity (95% CI)	Specificity (95% CI)
eADC map	CON	0.92 (0.87–0.97)	0.92 (0.86–0.98)	0.91 (0.82–0.97)	0.84 (0.79–0.88)
	CON + WT	0.96 (0.92–0.99)	0.97 (0.93–0.99)	0.94 (0.88–0.98)	0.91 (0.83–0.97)
T_1_Gd	CON	0.89 (0.83–0.91)	0.91 (0.83–0.97)	0.91 (0.85–0.98)	0.79 (0.74–0.82)
	CON + WT	0.94 (0.89–0.96)	0.97 (0.92–0.99)	0.97 (0.91–0.98)	0.92 (0.87–0.96)
ADC map	CON	0.86 (0.81–0.90)	0.91 (0.82–0.96)	0.87 (0.82–0.91)	0.81 (0.78–0.85)
	CON + WT	0.91 (0.86–0.95)	0.94 (0.89–0.98)	0.92 (0.83–0.97)	0.91 (0.87–0.93)
T_1_	CON	0.82 (0.79–0.86)	0.89 (0.85–0.92)	0.61 (0.57–0.71)	0.82 (0.79–0.86)
	CON + WT	0.91 (0.83–0.97)	0.93 (0.86–0.98)	0.81 (0.77–0.84)	0.91 (0.84–0.96)
T_2_‐FLAIR	CON	0.83 (0.79–0.88)	0.89 (0.86–0.92)	0.87 (0.81–0.91)	0.82 (0.80–0.85)
	CON + WT	0.86 (0.80–0.89)	0.91 (0.87–0.94)	0.94 (0.87–0.97)	0.94 (0.87–0.98)

## 4 Discussion

While the development of non‐invasive, accurate methods for glioma grading is a recognized priority, the potential of wavelet‐based radiomics from specific MRI image weights, such as DWI (ADC maps and eADC maps), remains underexplored. The present study was therefore conducted to systematically assess the contribution of wavelet features extracted from DWI MRI (ADC and eADC maps) and conventional structural MRI to ML‐based glioma HGG/LGG classification. To our knowledge, this work represents the first investigation to specifically utilize wavelet‐transformed eADC features for this purpose, thereby addressing a gap in the existing literature.

Performance evaluation based on AUC, sensitivity, specificity, accuracy, and ROC analysis demonstrated that all fine‐tuned ML classifiers trained with DWI (ADC and eADC maps; Figure 4) radiomics features significantly outperformed those utilizing structural MRI radiomics features for glioma HGG/LGG classification. The performance values of different trained MLs with structural MRI images are in good agreement with previous studies [12, 13, 14, 15]. A possible explanation for these results may be related to the physiological data in DWI images. DWI image contrast and data provide information about the water molecule's motility and diffusion coefficient in the tissue [23]. On the other hand, structural MRI images, such as T_1_ and T_2_‐FLAIR, provide structural data and limited physiological details. As reported by previous studies [11, 12], the evidence we found points to the superiority of DWI over structural MRI images for glioma grade classification with Radiomics analysis.

The comparative analysis within this study demonstrated that the fine‐tuned RF classifier, when applied to radiomics features from T_1_Gd and eADC maps, achieved superior performance in glioma HGG/LGG classification. This model yielded the highest AUC, sensitivity, specificity, and accuracy compared to all other fine‐tuned classifiers trained on different MRI weightings evaluated in this work. Interestingly, T_1_Gd image weight requires injecting a gadolinium‐based contrast agent, while DWI is a non‐invasive imaging protocol, which is one of the advantages of DWI over T_1_Gd. Consequently, based on its strong standalone performance and inherent clinical safety, the use of DWI‐derived radiomic features, particularly from quantitative maps such as ADC and eADC maps, is recommended by this study for further development and potential translation into a non‐invasive clinical tool for glioma grade classification.

This study demonstrates that, within a radiomics and ML framework, eADC maps provide more discriminative information for glioma grade HGG/LGG classification than standard ADC maps. This result can be attributed to the conventional ADC map being derived from the logarithm of the ratio S_DWI_/S_0_, whereas the eADC map is calculated directly from the ratio S_DWI_/S_0_ without logarithmic transformation [4, 24]. The use of logarithmic transformation in ADC map calculations diminishes certain contrast differences and information, rendering it less effective for glioma grading [4, 25].

Among all ML algorithms evaluated in this study, the fine‐tuned RF classifier consistently demonstrated superior performance for glioma grade classification. The ensemble‐based nature of RF enables effective handling of high‐dimensional radiomics features while reducing the risk of overfitting, resulting in stable and reliable classification performance. It should be noted that all ML models employed in this study were fine‐tuned to achieve optimal performance. These findings are consistent with previous studies reporting the advantages of RF in medical imaging and pattern recognition tasks [26]. Furthermore, comparison of parameters such as the AUC, ACC, sensitivity, and specificity obtained in the present study with those reported in prior investigations (Table 5) demonstrates comparable or improved performance. Notably, the results derived from diffusion‐weighted imaging, structural MRI sequences, and contrast‐enhanced T_1_‐weighted images are in agreement with existing literature, further supporting the effectiveness of the proposed radiomics‐based RF approach for glioma grade classification.

**Table 5:** Performance of previous studies for glioma grade classification with machine learning and radiomics analysis.

Authors	Used image data set	ML algorithm	AUC	ACC %
Cho et al. [14]	BraTs data set (T_1_, T_2_‐FLAIR, T_1_Gd, T_2_)	LR SVM RF	0.9 0.88 0.92	0.88 0.88 0.88
Tian et al. [12]	T_1_, T_2_, DWI, ASL	SVM	0.987	96.8
Cho et al. [15]	BraTs data set (T_1_, T_2_‐FLAIR, T_1_Gd, T_2_)	LR	0.88	89
Zhou et al. [13]	T_1_Gd	LR	0.95	84.8
Ouerghi et al. [27]	BraTs data set (T_1_, T_2_‐FLAIR, T_1_Gd, T_2_)	KNN MLP LB RF	0.99 0.902 0.982 0.995	94.6 83.8 93.8 96.5
Chen et al. [28]	T_1_, T_2_‐FLAIR, T_1_Gd, T_2_	SVM	0.93	91.27
Wu et al. [29]	T_1_, T_2_‐FLAIR, T_2_	LR	0.96	91.3
Chiu et al. [30]	T_1_Gd, T_2_, T_2_‐FLAIR, ADC map	KNN NB RF	0.6 0.96 0.96	55.9 80.6 90.4
Hashido et al. [31]	ADC map, perfusion	RF SVM	0.917 0.717	NA
Takahashi et al. [11]	ADC maps	LR SVM	0.74 0.58	63.6 81.8

Abbreviations: ACC, Accuracy; ADC, apparent diffusion coefficient; AUC, Area under the curve; ASL, Arterial spin labeling; DWI, Diffusion‐weighted imaging; KNN, K‐nearest neighbors; LR, linear regression; ML, machine learning; MLP, Multi‐layer Perceptron; NA, not available; NB, Naïve Bayes; RF, random forest; SVM, support vector machines.

The most significant finding of this study is that incorporating Wavelet‐based radiomic features significantly enhances the performance of ML algorithms in classifying glioma grades into the LGGs and HGGs. Notably, this improvement was observed across multiple classifiers, including KNN, DT, NB, LR, SVM, MLP, and RF, which used in this study. These results align with prior research [10, 32, 33] further supporting the utility of Wavelet‐based radiomics as a robust approach for glioma grading. The underlying mechanism for this enhancement likely stems from the ability of the Wavelet transform to decompose images into multiple sub‐bands, thereby extracting supplementary textural information related to tumor heterogeneity that is not readily captured by conventional feature sets. This multi‐resolution analysis provides a more comprehensive characterization of the tumor's spatial complexity. Overall, the current study demonstrated that fine‐tuned RF models trained on conventional and wavelet‐based radiomics features extracted from eADC maps achieved the highest performance for low‐grade versus high‐grade glioma classification.

This study has several important limitations that must be acknowledged. First, the single‐center, single‐scanner design, while ensuring technical consistency, inherently introduces scanner bias and limits the generalizability of our findings to other institutions with different MRI hardware and protocols. Second, the reliance on manual segmentation for VOI creation, though performed by experts, is subject to inter‐rater variability and is time‐consuming; this variability can influence feature stability. Although we employed a strict patient‐wise data splitting strategy to mitigate the risk of data leakage, the performance of our models requires rigorous external validation. The most critical next step is to evaluate our pipeline on an independent, multi‐institutional cohort to assess its true robustness and clinical transportability. Furthermore, our classification target was traditional histological grade (LGG/HGG). The relationship between the identified radiomic signatures and the now clinically essential molecular subtypes (e.g., IDH mutation status) remains unexplored and represents a vital direction for future work to enhance diagnostic relevance. Furthermore, as described in Section 2.6, while we used a held‐out test set for final evaluation, our development pipeline performed feature selection (LASSO) on the entire training set prior to hyperparameter tuning via cross‐validation. This sequential approach, rather than a fully nested cross‐validation framework, may introduce optimism bias in the model selection process and could inflate the perceived importance of the selected features. Future studies employing nested cross‐validation are needed to validate the stability and generalizability of the identified radiomic signature.

For future research, several directions may further enhance the robustness and clinical applicability of radiomics‐based glioma grading. Previous studies have demonstrated that radiomics features extracted from the peritumoral edema region can provide valuable information for glioma grade classification [2, 34] Building on these findings, future investigations could extend the current methodology by incorporating radiomics features derived from peritumoral edema using eADC maps to further improve grading performance. In addition, manual tumor segmentation and mask generation in the present study were time‐consuming and operator‐dependent. Future work may benefit from the integration of automated deep learning–based segmentation approaches, such as U‐Net and its variants, to enable faster, more reproducible, and fully automated tumor delineation. Moreover, the inclusion of radiomics features from additional MRI sequences, such as susceptibility‐weighted imaging (SWI), perfusion imaging, and other advanced MRI modalities, may provide complementary tumor information and further improve the accuracy of glioma grade classification models.

## 5 Conclusion

This study aimed to investigate the impact of incorporating wavelet‐based radiomics features on the performance of multiple ML models for glioma grade classification using diffusion‐weighted imaging–derived maps (ADC and eADC), conventional structural MRI sequences (T_1_‐weighted and T_2_‐FLAIR), and contrast‐enhanced T_1_‐weighted images (T_1_Gd). The findings demonstrate that the inclusion of wavelet‐based features consistently enhanced the classification performance of all evaluated ML algorithms, highlighting the added value of multi‐scale textural information in radiomics‐based glioma grading. Furthermore, comparative analysis across different MRI sequences revealed that fine‐tuned Random Forest models trained on radiomics features extracted from eADC maps achieved the highest performance for distinguishing low‐grade from HGGs. Collectively, these results suggest that combining eADC map‐based radiomics with wavelet feature analysis and Random Forest classification offers a robust, accurate, and non‐invasive approach for glioma grade assessment, with potential applicability as a fast and reliable tool to support clinical decision‐making.

## Author Contributions

**Amir Khorasani:** conceptualization, methodology, validation, investigation, resources, data curation, writing – original draft, writing – review and editing, software, formal analysis, visualization. **Daryoush Shahbazi‐Gahrouei:** conceptualization, investigation, resources, writing – review and editing, supervision, project administration, funding acquisition, visualization.

## Disclosure

All authors have read and approved the final version of the manuscript. Daryoush Shahbazi‐Gahrouei had full access to all of the data in this study and takes complete responsibility for the integrity of the data and the accuracy of the data analysis.

## Ethics Statement

The Local Research Ethics Board, the Isfahan University of Medical Sciences, Isfahan, Iran, research ethics committee approved the study with ID: IR. ARI. MUI. REC.1403.054.

## Conflicts of Interest

The authors declare no conflicts of interest.

## Transparency Statement

The Daryoush Shahbazi‐Gahrouei affirms that this manuscript is an honest, accurate, and transparent account of the study being reported; that no important aspects of the study have been omitted; and that any discrepancies from the study as planned (and, if relevant, registered) have been explained.

## Supporting information


Supporting File

## Data Availability

The data that support the findings of this study are available from the corresponding author upon reasonable request.
