# Supplementary material for: Glioma Grade Classification Using Machine Learning and MRI Radiomics: A Single‐Center Prospective Study Comparing Original and Wavelet‐Transformed Features From Anatomical, Diffusion‐Weighted, and Post‐Contrast Imaging
Source: Health Sci Rep. 2026 Aug 11;9(8):e72995. doi: 10.1002/hsr2.72995 (PMC13462304; doi:10.1002/hsr2.72995)
Supplement: Supplementary file 1 [file HSR2-9-e72995-s001.docx]

Supplementary 1- Feature ‘Class’ and ‘Names’ used in this study. Abbreviation: Gray-level co-occurrence matrix (GLCM), Gray-level dependence matrix (GLDM), Gray-level run length matrix (GLRLM), Gray-level size-zone matrix (GLSZM), Neighboring gray-tone difference matrix (NGTDM).

| Feature Class | Feature Name |
| --- | --- |
| shape | Elongation |
| shape | Flatness |
| shape | LeastAxisLength |
| shape | MajorAxisLength |
| shape | Maximum2DDiameterColumn |
| shape | Maximum2DDiameterRow |
| shape | Maximum2DDiameterSlice |
| shape | Maximum3DDiameter |
| shape | MeshVolume |
| shape | MinorAxisLength |
| shape | Sphericity |
| shape | SurfaceArea |
| shape | SurfaceVolumeRatio |
| shape | VoxelVolume |
| firstorder | 10Percentile |
| firstorder | 90Percentile |
| firstorder | Energy |
| firstorder | Entropy |
| firstorder | InterquartileRange |
| firstorder | Kurtosis |
| firstorder | Maximum |
| firstorder | MeanAbsoluteDeviation |
| firstorder | Mean |
| firstorder | Median |
| firstorder | Minimum |
| firstorder | Range |
| firstorder | RobustMeanAbsoluteDeviation |
| firstorder | RootMeanSquared |
| firstorder | Skewness |
| firstorder | TotalEnergy |
| firstorder | Uniformity |
| firstorder | Variance |
| glcm | Autocorrelation |
| glcm | ClusterProminence |
| glcm | ClusterShade |
| glcm | ClusterTendency |
| glcm | Contrast |
| glcm | Correlation |
| glcm | DifferenceAverage |
| glcm | DifferenceEntropy |
| glcm | DifferenceVariance |
| glcm | Id |
| glcm | Idm |
| glcm | Idmn |
| glcm | Idn |
| glcm | Imc1 |
| glcm | Imc2 |
| glcm | InverseVariance |
| glcm | JointAverage |
| glcm | JointEnergy |
| glcm | JointEntropy |
| glcm | MCC |
| glcm | MaximumProbability |
| glcm | SumAverage |
| glcm | SumEntropy |
| glcm | SumSquares |
| gldm | DependenceEntropy |
| gldm | DependenceNonUniformity |
| gldm | DependenceNonUniformityNormalized |
| gldm | DependenceVariance |
| gldm | GrayLevelNonUniformity |
| gldm | GrayLevelVariance |
| gldm | HighGrayLevelEmphasis |
| gldm | LargeDependenceEmphasis |
| gldm | LargeDependenceHighGrayLevelEmphasis |
| gldm | LargeDependenceLowGrayLevelEmphasis |
| gldm | LowGrayLevelEmphasis |
| gldm | SmallDependenceEmphasis |
| gldm | SmallDependenceHighGrayLevelEmphasis |
| gldm | SmallDependenceLowGrayLevelEmphasis |
| glrlm | GrayLevelNonUniformity |
| glrlm | GrayLevelNonUniformityNormalized |
| glrlm | GrayLevelVariance |
| glrlm | HighGrayLevelRunEmphasis |
| glrlm | LongRunEmphasis |
| glrlm | LongRunHighGrayLevelEmphasis |
| glrlm | LongRunLowGrayLevelEmphasis |
| glrlm | LowGrayLevelRunEmphasis |
| glrlm | RunEntropy |
| glrlm | RunLengthNonUniformity |
| glrlm | RunLengthNonUniformityNormalized |
| glrlm | RunPercentage |
| glrlm | RunVariance |
| glrlm | ShortRunEmphasis |
| glrlm | ShortRunHighGrayLevelEmphasis |
| glrlm | ShortRunLowGrayLevelEmphasis |
| glszm | GrayLevelNonUniformity |
| glszm | GrayLevelNonUniformityNormalized |
| glszm | GrayLevelVariance |
| glszm | HighGrayLevelZoneEmphasis |
| glszm | LargeAreaEmphasis |
| glszm | LargeAreaHighGrayLevelEmphasis |
| glszm | LargeAreaLowGrayLevelEmphasis |
| glszm | LowGrayLevelZoneEmphasis |
| glszm | SizeZoneNonUniformity |
| glszm | SizeZoneNonUniformityNormalized |
| glszm | SmallAreaEmphasis |
| glszm | SmallAreaHighGrayLevelEmphasis |
| glszm | SmallAreaLowGrayLevelEmphasis |
| glszm | ZoneEntropy |
| glszm | ZonePercentage |
| glszm | ZoneVariance |
| ngtdm | Busyness |
| ngtdm | Coarseness |
| ngtdm | Complexity |
| ngtdm | Contrast |
| ngtdm | Strength |
